# When Less Is More: Downsizing, Sense of Place, and Well-Being in Late Life

**DOI:** 10.1093/geroni/igaa057.1423

**Published:** 2020-12-16

**Authors:** Kyrsten Costlow, Shinae Choi, Beverly Roskos, Patricia Parmelee

**Affiliations:** 1 The University of Alabama, Tuscaloosa, Alabama, United States; 2 University of Alabama, Tuscaloosa, Tuscaloosa, Alabama, United States; 3 University of Alabama, Tuscaloosa, Alabama, United States

## Abstract

The present study aimed to investigate the decision-making process and outcomes associated with downsizing to a smaller home in late life. Older adults who had downsized in the past year (n = 68) completed self-report measures of push-pull factors driving the decision to move, relocation controllability, sense of place (SOP), move satisfaction, and psychological well-being. It was hypothesized that the relation between push-pull factors and relocation outcomes (i.e., move satisfaction and psychological well-being) would be serially mediated by control and SOP. Haye’s PROCESS macro was used to test serial multiple mediator models for each of the relocation outcomes. Placing greater importance on push relative to pull factors was associated with lower levels of well-being in three domains: environmental mastery (b = -5.52, p = .002), purpose in life (b = -3.94, p = .01), and self-acceptance (b = -3.61, p = .007). Results of serial mediation analyses suggested that older adults whose downsizing decisions were more strongly influenced by push factors felt less control over relocation, found it more difficult to develop SOP in the new home, and, in turn, experienced lower levels of psychological well-being and move satisfaction. These findings can be used to inform older adults’ downsizing decisions and develop supports for relocating older adults.

